# A preliminary retrospective study of the safety of Vancomycin area under the curve in patients treated with concomitant use of Vancomycin and gentamicin

**DOI:** 10.1186/s40780-025-00438-1

**Published:** 2025-04-14

**Authors:** Hirokazu Nakayama, Yoshitsugu Nakamura, Masayo Tanaka

**Affiliations:** 1 Department of Pharmacy, NTT Medical Center Tokyo, 5-9-22 Higashi-gotanda, Shinagawa-ku, Tokyo, 141-8625 Japan; 2 Department of Cardiovascular Surgery, Chiba-Nishi General Hospital, Chiba, Japan

**Keywords:** Vancomycin, Gentamicin, Infective endocarditis, Acute kidney injury, Therapeutic drug monitoring

## Abstract

**Background:**

Despite numerous studies on safety, acute kidney injury (AKI) caused by vancomycin and/or gentamicin remains a persistent medical issue. However, it remains unclear whether vancomycin AUC below 600 mg·h/L in combination with gentamicin trough level at least below 2 µg/mL are reliable indices to reduce the risk of AKI in patients treated with concomitant vancomycin and gentamicin.

**Objective:**

The aim was to elucidate the pharmacokinetic factors associated with AKI development in patients receiving concomitant use of vancomycin and gentamicin in the setting of therapeutic drug monitoring (TDM).

**Methods:**

A retrospective study was conducted in 15 patients treated with concomitant vancomycin and gentamicin with TDM. The patients were classified into AKI group and no-AKI group. Vancomycin area under the curve (AUC), gentamicin trough levels, and duration of concomitant duration of vancomycin and gentamicin were investigated.

**Results:**

Six (40%) of 15 patients developed AKI during the study period. In AKI group (*n* = 6), vancomycin AUC was significant higher [median (range) 561 (543‒712) mg·h/L compared to no-AKI group (*n* = 9), 380 (185‒600) mg·h/L, *p* = 0.026)], although no significant differences in gentamicin trough level and duration of concomitant vancomycin and gentamicin treatment were found between the two groups. Receiver operating characteristic analysis showed that the best cut-off vancomycin AUC for predicting AKI was 523 mg·h/L, with AUC of 0.852, sensitivity of 1.000 and specificity of 0.778 (*p* = 0.025).

**Conclusions:**

In patients treated with concomitant vancomycin and gentamicin with trough level below 1–2 µg/mL, vancomycin AUC 530 − 600 mg·h/L is associated with AKI risk.

**Supplementary Information:**

The online version contains supplementary material available at 10.1186/s40780-025-00438-1.

## Introduction

Despite numerous studies on safety, acute kidney injury (AKI) caused by vancomycin and/or gentamicin remains a persistent medical issue [1, 2]. The revised consensus guideline for therapeutic drug monitoring (TDM) of vancomycin recommends dosing guided by area under the curve (AUC), 400–600 mg·h/L to minimize the risk of vancomycin-associated nephrotoxicity [3]. In addition, gentamicin trough concentration at least less than 2 µg/mL, but preferably less than 0.5–1 µg/mL and/or the once daily administration have proven to reduce AKI. However, in the recent meta-analysis, gentamicin trough concentration less than 2 µg/mL was linked to lower frequency of nephrotoxicity [4, 5]. Furthermore, reduction in endogenous creatinine clearance reduces was associated with the length of gentamicin treatment [6].

However, it remains unclear whether vancomycin AUC less than 600 mg·h/L in combination with gentamicin trough levels less than 1–2 µg/mL are reliable indices to reduce the risk of AKI in patients treated with concomitant vancomycin and gentamicin, even when treatment is conducted with adherence to TDM. The aim of this study was to elucidate the pharmacokinetic factors associated with AKI development in patients receiving concomitant use of vancomycin and gentamicin in the setting of TDM.

## Methods

### Patient enrollment

A retrospective medical chart review was conducted at NTT Medical Center Tokyo between December 1, 2000, and July 31, 2023. In this study, patients who (i) were aged 18 years or older, (ii) had received concomitant use of intravenous vancomycin plus gentamicin for at least 2 consecutive days, (iii) had baseline serum creatinine level of 1.6 mg/dL or lower according to a previous report with modification [7], and (iv) received TDM of vancomycin and gentamicin in both, were included. In addition, patients with median gentamicin trough levels 2 µg/mL or more during the study period, were excluded, to mitigate the influence of nephrotoxicity caused by higher gentamicin exposure in this analysis according to a previous report [5]. The study protocol was approved by the Ethics Committee of NTT Medical Center Tokyo (Approval Number: 00 200022825-01) prior to the study. Data collection, assessment of qSOFA score, vancomycin administration and pharmacokinetic analysis, gentamicin administration, vancomycin and gentamicin assay were described in supplemental materials.

### Clinical outcomes, AKI definition, and classification of patients

Clinical outcomes were defined as 30- and 90-day mortality, clinical failure, and AKI.

Clinical failure was defined as patients who received add-on treatment for infectious diseases with concomitant use of vancomycin and gentamicin, or who had clinical signs and/or symptoms of exacerbation infectious diseases at least after 3 days of the commencement of concomitant use vancomycin and gentamicin according to a previous report with modification [9].

AKI was defined as an increase in serum creatinine of 0.5 mg/dL or by 50% in at least 2 consecutive measurements between the first dose and 72 h after the last dose of vancomycin [7, 8]. Patients were divided into AKI group and no-AKI group. Vancomycin and gentamicin concentrations before the onset of AKI in AKI group, and during the entire treatment duration in no-AKI group were analyzed [7, 8]. In patients who received multiple TDM assessments during the study period, the median value was used for analysis.

### Statistical analysis

Statistical comparisons between two groups were performed using Mann–Whitney U test for continuous variable and Fisher exact test for categorical data, respectively. Subsequently, receiver operating characteristic (ROC) analysis was performed to determine the cut-off for discrimination. A *p* value less than 0.05 was considered statistically significant.

## Results

We retrieved the clinical data from 42 patients who received concomitant intravenous vancomycin and gentamicin treatment during the study period. Among them, 22 patients who did not received TDM of gentamicin, 3 patients who received TDM of vancomycin or gentamicin after onset of AKI, 1 patient with baseline serum creatinine higher than 1.6 mg/dL, and 1 patient with median gentamicin trough level more than 2 μg/mL were excluded. Eventually, a total of 15 patients were eligible for this analysis. The subjects comprised 12 males and 3 females with median (range) age of 66 (21–82) years. Six (40%) of 15 patients developed AKI during the study period. While one patient who had unstable kidney function, elevating in serum creatinine of 0.5 mg/dL within 1 day before the day of commencement of concomitant vancomycin and gentamicin treatment was included, no increase in serum creatinine was observed during the period of the concomitant treatment of the two drugs. In addition, no patients were administered concomitant piperacillin-tazobactam treatment.

The patients were divided into AKI group (*n* = 6) and no-AKI group (*n* = 9) (Table 1). While there was a significant difference between the two groups in infective endocarditis as main diagnosis, no significant differences between the two groups were observed in vancomycin and gentamicin daily dose, as well as age, other comorbidities (including chronic kidney disease (CKD), kidney function, ejection fraction), frequency of qSOFA score 2 or more, complete blood counts, and concomitant nephrotoxic agents (Table 1).

**Table 1:** Demographic and relevant clinical characteristics, and dosage of VCM and GM

Variable	AKI Group (*n* = 6)	no-AKI Group (*n* = 9)	*P*-value
Age (years)	70 (54–81)	66 (21–82)	0.388
Sex male -no. (%)	5 (83)	7 (78)	1.000
Body weight (kg)	64 (48–76)	59 (40–79)	0.388
Main diagnosis - no. (%)			
Native valve IE / Prosthetic valve IE	4 (67) / 2 (33)	3 (30) / 1 (11)	0.044
Sepsis	0 (0)	2 (22)	0.486
Bacteremia	0 (0)	2 (22)	0.486
Others ^1)^	0 (0)	1 (11)	1.000
Causative pathogens no. (%)			
*MRSA*	0 (0)	2 (22)	0.486
*Staphylococcus epidermidis*	1 (17)	0 (0)	0.400
*Enterococcus species*	1 (17)	2 (22)	1.000
*Streptococcus species*	2 (33)	2 (22)	1.000
Others or not identified	2 (33)	3 (33)	1.000
Comorbidities no. (%)			
Valvular disease	3 (50)	3 (33)	0.622
Arrhythmia	1 (17)	1 (11)	1.000
Cerebrovascular disease	0 (0)	3 (33)	0.229
Hypertension	3 (50)	1 (11)	0.235
CKD	1 (17)	0 (0)	0.400
Hematological malignancy	1 (17)	1 (11)	1.000
Solid tumor	0 (0)	2 (22)	0.486
qSOFA score 2 or higher / others - no. (%) ^2)^	0 (0) / 6 (100)	1 (11) / 8 (89)	1.000
Ejection fraction (%)	73 (66–81)	70 (38–85)	0.776
Heart valve surgery no. (%)	1 (17)	1 (11)	1.000
Baseline biochemistry and complete blood counts			
Albumin (g/dL)	2.8 (2.0–3.6)	2.7 (2.2–4.2)	0.767
Blood urea nitrogen (mg/dL)	18.3 (8.6–24.5)	13.2 (7.8–47.8)	0.480
Creatinine (mg/dL)	0.84 (0.35–1.29)	0.65 (0.49–1.56)	0.637
CLcr (mL/min)	68 (58–136)	81 (31–159)	0.556
eGFR (mL/min/1.73m^2^)	68 (47–142)	93 (36–147)	0.814
Hemoglobin (g/dL)	11.2 (7.1–15.6)	10.0 (7.2–13.7)	0.529
VCM dose (mg/kg/day)	26.8 (16.5–38.9)	25.4 (10.6–46.2)	0.607
GM dose (mg/kg/day)	2.0 (1.6–2.5)	2.0 (0.8–4.6)	0.864
GM once daily administration no. (%)	3 (50)	4 (44)	1.000
Duration until AKI onset after the commencement of VCM plus GM therapy (day)	12 (7–22)	NA	NA
Duration until AKI onset after the commencement of VCM therapy	14 (7–24)	NA	NA
Frequency of VCM TDM during the study period (day)	4 (1–4)	2 (1–5)	0.251
Median duration until TDM after the commencement of VCM (day)	9 (5–14)	5 (4–7)	0.097
Clinical outcomes ^3)^			
30-day mortality no. (%)	1 (17)	0 (0)	0.429
90-day mortality no. (%)	3 (50)	1 (13)	0.245
Clinical failure no. (%)	2 (33) ^4)^	3 (38) ^5)^	1.000
Concomitant nephrotoxic agents no. (%)			
ACEIs/ARBs	1 (17)	0 (0)	0.400
NSAIDs	0 (0)	1 (11)	1.000
Diuretics	1 (17)	3 (33)	0.604
Immunosuppressant	0 (0)	1 (11)	1.000
Concomitant other antimicrobial agents no. (%)			
Cephem antimicrobial agents ^6)^	1 (17)	3 (33)	0.604
Meropenem	0 (0)	2 (22)	0.486

Data are expressed as median (range) or number of subjects (percent)

Abbreviations: ACEIs/ARBs; angiotensin converting enzyme inhibitors or angiotensin receptor blockers, CKD; chronic kidney disease, CLcr, creatinine clearance, CFPM; cefepime, CTRX; ceftriaxone, GM; gentamicin, IE; infective endocarditis, *MRSA*; *methicillin-resistant staphylococcus aureus*, NA; not available, NSAIDs; non-steroidal anti-inflammatory drugs, qSOFA; quick sequential organ failure assessment, TDM; therapeutic drug monitoring, VCM; vancomycin

^1^Infection after cord blood transplantation due to hematological malignancy in 1 patient were included

^2^Data of respiratory rate were not available in 1 patient in AKI group and in 3 patients in no-AKI group. These 4 patients had systolic blood pressure higher than 100 mmHg and Glasgow Coma scale score of 15. Eventually, the qSOFA scores were calculated as 0 or 1

^3^One patient in no-AKI group was censored due to another institute

^4^Ineffectiveness of vancomycin in 2 patients

^5^Relapse of infectious diseases in 2 patients, delayed hypersensitivity reaction to vancomycin in 1 patient, relapse of febrile neutropenia in 1 patient

^6^CTRX was administered in 1 in AKI group. CTRX was administered in 2, CFPM was administered in 1 in no-AKI group

**Fig. 1:**
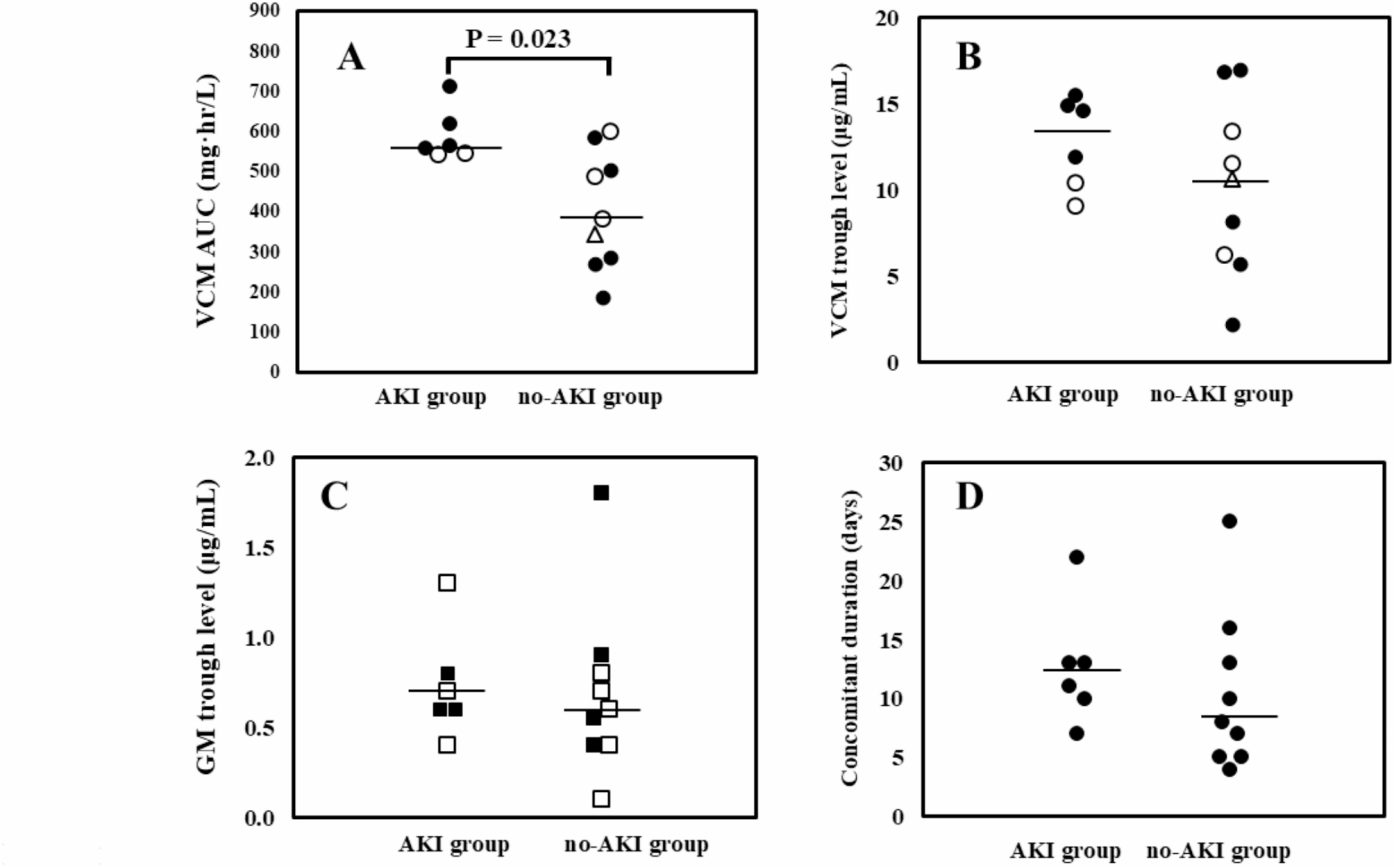
**A**: Comparison of VCM AUC between AKI group and no-AKI group; median (range): 561 (543‒712) mg·h/L and 380 (185‒600) mg·h/L, respectively. Solid bars denote median values. Open circles show patients who developed clinical failure. Open triangle shows one patient whose record regarding the outcome of infectious disease was not available due to transfer to another institute. **B**: Comparison of VCM trough levels between AKI group and no-AKI group; median (range): 13.3 (9.1‒15.5) µg/mL vs. 10.6 (2.2‒17.0) µg/mL, respectively. Open circles show patients who developed clinical failure. Open triangle shows one patient whose record regarding the outcome of infectious disease was not available due to transfer to another institute. **C**: Comparison of GM trough levels between AKI group and no-AKI group; median (interquartile range): 0.7 (0.4‒1.3) µg/mL and 0.6 (0.1‒1.8) µg/mL, respectively. Solid bars denote median values. Closed squares show patients treated with once daily administration of GM. Open squares show patients treated with twice or three times daily administration of GM. **D**: Comparison of concomitant duration of vancomycin and gentamicin between AKI group and no-AKI group; median (range): 12 (7‒22) days and 8 (4‒25) days, respectively. Solid bars denote median values. Abbreviations: AKI; acute kidney injury, AUC; area under the curve, GM; gentamicin, mono; monotherapy, VCM; vancomycin

**Fig. 2:**
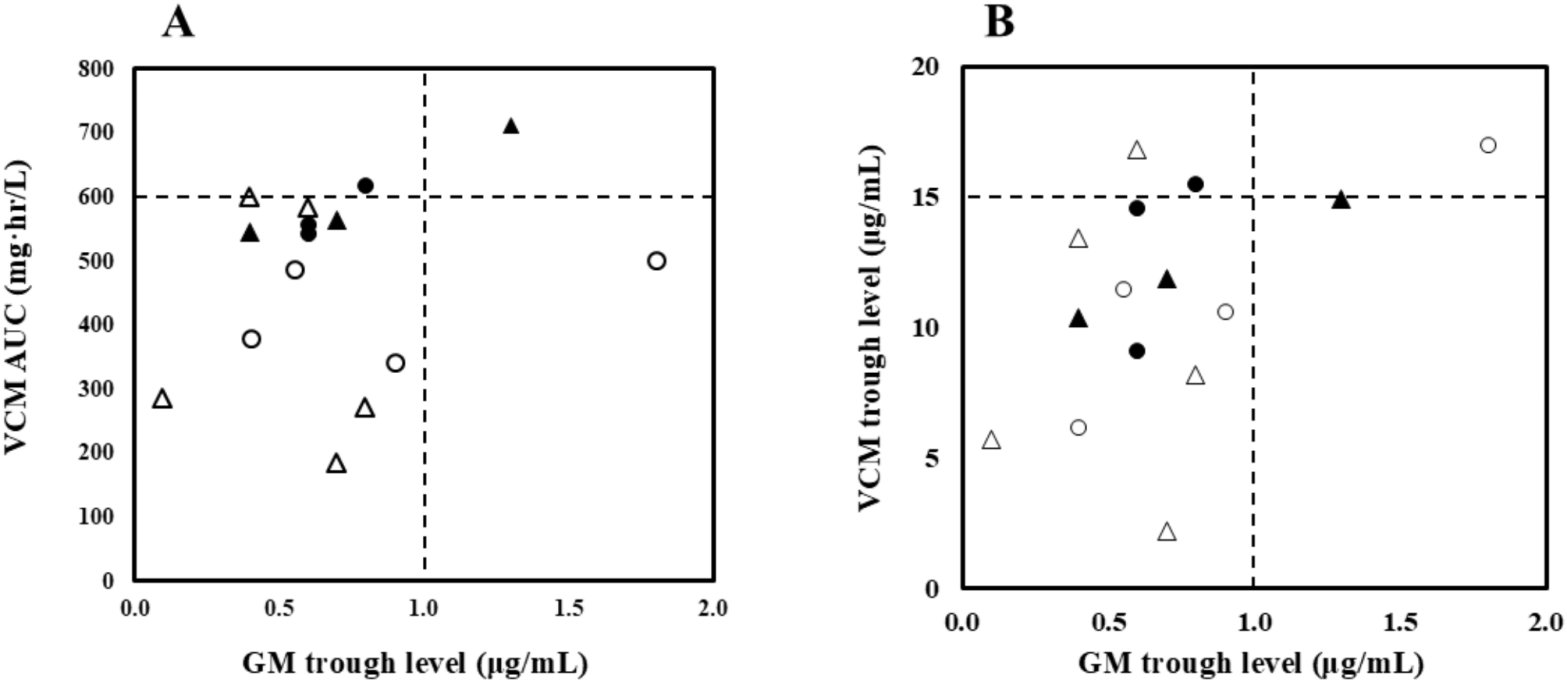
**A**: Relation between VCM AUC and GM trough level. Horizontal dot line shows the guideline-recommended threshold of VCM AUC 600 mg∙h/mL. Vertical dot line shows threshold of GM level 1 µg/mL. **B**: Relation between VCM trough level and GM trough level. Horizontal dot line shows the guideline-recommended threshold of VCM trough level 15 µg/mL. Vertical dot line shows threshold of GM level 1 µg/mL. Closed circles show patients who developed AKI and treated with once daily administration of GM. Closed triangles show patients who developed AKI and treated with twice or three times daily administration of GM. Open circles show patients who did not develop AKI and treated with once daily administration of GM. Open triangles show patients who did not developed AKI and treated with twice or three times daily administration of GM. Abbreviations: AKI; acute kidney injury, AUC; area under the curve, GM; gentamicin, mono; monotherapy, VCM; vancomycin

There was a significant difference between the two groups in vancomycin AUC [AKI group vs. no-AKI group: 561 (543‒712) mg·h/L vs. 380 (185‒600) mg·h/L, *p* = 0.026] (Fig. 1A). By contrast, no significant differences between AKI group and no-AKI group were observed 13.3 (9.1‒15.5) µg/mL vs. 10.6 (2.2‒17.0) µg/mL in trough vancomycin concentration (*p* = 0.388) and 0.7 (0.4‒1.3) µg/mL vs. 0.6 (0.1‒1.8) µg/mL] in trough gentamicin concentration (*p* = 0.689) (Fig. 1BC). Among 6 patients who developed AKI, serum creatinine increased 1.5 to 1.9-fold in 5 patients and 2 to 3-fold in 1 patient. In addition, no significant difference was observed between the two groups in the length of concomitant treatment of vancomycin and gentamicin [AKI group vs. no-AKI group: 12 (7‒22) days vs. 8 (4‒25) days (*p* = 0.286) (Fig. 1D)]. In addition, two patients had vancomycin AUC more than 600 mg·h/L, and one of two patients did the value with gentamicin trough level more than 1 µg/mL in AKI group. In no-AKI group, 1 patient had vancomycin AUC 600 mg·h/L and 1 patient did gentamicin trough level more than 1 µg/mL (Fig. 2AB). In the calculation of vancomycin AUC, one patient in no-AKI group was assessed using only one sampling point. In addition, the frequency of TDM was 4 (1‒4) times in AKI group vs. 2 (1‒5) times in no-AKI group.

ROC analysis showed that the best cut-off vancomycin AUC for predicting AKI was 523 mg·h/L, with AUC of 0.852, sensitivity of 1.000 and specificity of 0.778 (*p* = 0.025).

The numbers of patients associated with 30-day, and 90-days mortality were 1 (17%) in AKI group vs. 0 (0%) in no-AKI group (*p* = 0.429), and 3 (50%) in AKI group vs. 1 (13%) in no-AKI group (*p* = 0.245), respectively. In the present study, while clinical failure was observed in 5 patients who were ineffectiveness of concomitant vancomycin and gentamicin treatment in 2 patients, and relapsed infectious diseases or febrile neutropenia in 3 patients, respectively, no difference between the two groups was observed (*p* = 1.000) (Table 1). In an additional analysis using groups according to the threshold of vancomycin AUC, no differences in 30-day mortality, 90-day mortality, and clinical failure were observed. The proportion of AKI development was 75% in vancomycin AUC more than 523 mg·h/L group vs. 0% vancomycin AUC 523 mg·h/L or less group (*p* = 0.007) (Table 2).

**Table 2:** Clinical outcomes between two groups divided by Vancomycin AUC threshold

Variable	Vancomycin AUC > 523 mg·h/L (*n* = 8)	Vancomycin AUC ≤ 523 mg·h/L (*n* = 7)	*P*-value
30-day mortality no. (%) ^1)^	1 (13)	0 (0)	1.000
90-day mortality no. (%) ^1)^	3 (38)	1 (17)	0.580
Clinical Failure no. (%) ^1)^	3 (38) ^2)^	2 (33) ^3)^	1.000
AKI no. (%)	6 (75)	0 (0)	0.007

Abbreviations: AKI; acute kidney injury, AUC; area under the curve

^1^One patient with vancomycin AUC ≤ 523 mg·h/L was censored due to another institute

^2^Ineffectiveness of vancomycin in 2 patients, and relapse of infectious diseases in 1 patient

^3^Relapse of infectious diseases in 1 patient, and relapse of febrile neutropenia in 1 patient

## Discussion

This preliminary study suggests that vancomycin AUC ranging 530–600 mg·h/L potentially leads to development of AKI in patients treated with concomitant vancomycin and gentamicin with trough level less than 1–2 µg/mL.

Particularly, the threshold of vancomycin AUC 523 mg·h/L, using ROC analysis, which was denoted lower than the value in the guideline [3], should be interpreted with caution. In the present study, no patient with approximately vancomycin AUC below 530 mg·h/L developed AKI. Nevertheless, we cannot categorically rule out the possibility that the cut-off value was estimated by chance due to obtained data from only 15 patients. However, vancomycin AUC between 500 and 600 mg·h/L was likely to increase the risk of AKI in critically ill patients and/or in patients with risk factors for nephrotoxicity, including concomitant use of vancomycin and nephrotoxins in recent reports [10, 11, 12, 13]. Our findings are concordant with the reports. Furthermore, in patients treated with concomitant vancomycin with AUC below 600 mg·h/L and piperacillin/tazobactam, a nephrotoxin, the higher frequency of AKI was observed compared to concomitant treatment of cefepime, no-nephrotoxin [14]. Since a cause of AKI development in patient treated with vancomycin combined with gentamicin is synergic nephrotoxic effect between the two agents [1], thus, the threshold of vancomycin AUC for nephrotoxicity may reduce due to nephrotoxin, such as gentamicin or piperacillin/tazobactam, exposure. No recommended index to reduce the risk of AKI is available for patients treated with concomitant aminoglycoside and vancomycin. We propose that vancomycin AUC 530 − 600 mg·h/L is associated with development of AKI, until further convincing data is available, particularly in patients treated with concomitant use of vancomycin and gentamicin.

While numerous potential risk factors of nephrotoxicity are ascertained, it remains uncertain whether the risk of AKI associated with use of vancomycin combined with gentamicin is due to severity of underlying illness [1]. In this regard, we assessed the systemic conditions using qSOFA score. While lower qSOFA scores less than 2 are associated with non-ICU admission [15], 14 of 15 patients had the scores lower than 2. In addition, cardiac function was preserved since ejection fraction was 70 (65 − 80) %. In the present study, hemodynamically stable patients may be enrolled at baseline.

The value of vancomycin AUC 400–600 mg·h/L is recommended as the range to prevent AKI [3]. In the present study, 4 of 6 patients developed AKI, despite vancomycin AUC 500 − 600 mg·h/L. In this regard, due to synergistic nephrotoxicity between two drugs, the threshold of vancomycin AUC for AKI may reduce. However, it is not clear whether synergistic aminoglycosides should be used for the entire duration of therapy. Therefore, concomitant gentamicin should be administrated prudently.

This study had a number of limitations in addition to those inherent to retrospective study with a small sample size. First, this small-scale single-center retrospective study did not allow multivariable analysis. Therefore, a definite conclusion cannot be depicted. Furthermore, caution should be exercised for generalizing our findings due to preliminary study design. In the present study, 6 of 10 patients with infective endocarditis developed AKI. Among them, 3 patients had 90-day mortality. In approximately 30% of IE patients, acute kidney injury (AKI) occurs during treatment due to various reasons including hemodynamic impairment, perioperative anemia, and administration of vancomycin and/or aminoglycoside [16, 17]. Previous reports suggest that impaired kidney function is a prognostic factor for both morbidity and mortality in patients with IE [18]. Second, in the present study, it would be difficult to assess the efficacy of vancomycin AUC with concomitant gentamicin, due to various reasons, including hematological stem cell transplantation, and exacerbation of primary disease. Reduced threshold of vancomycin AUC for prevention AKI development may lead to clinical failure due to subtherapeutic level of vancomycin AUC. Of note, the development of clinical failure in the treatment of concomitant use of vancomycin and gentamicin with adherence TDM was not associated with vancomycin AUC (Table 2). In the present study, while we consider that 6 patients developed clinical failure, there was only one patient who developed clinical failure. regardless of vancomycin AUC less than 400 mg·h/L. Indeed, the patient with vancomycin AUC 286 mg·h/L and gentamicin trough level 0.1 µg/mL was 21 years old, male. He might have argument renal clearance of vancomycin. In addition, since the patient with vancomycin AUC 185 mg·h/L and gentamicin trough level 0.7 µg/mL whose minimum inhibitory concentration (MIC) was 0.5 µg/mL, as a result, AUC/MIC was obtained as 390 mg·h/L, approximately recommended value. The role of vancomycin TDM is to prevent AKI as well as clinical failure in patients with subtherapeutic levels of vancomycin AUC. Therefore, the individual treatment with adherence to TDM to in patients treated with vancomycin and concomitant nephrotoxins is mandatory. Third, another limitation is with respect to assessment of baseline kidney function. While Lodise et al. adopted serum creatinine 2.0 mg/dL or lower at baseline as an inclusion criterion, the threshold could inadvertently include patients with CKD or a pre-existing AKI. Accordingly, the value of 1.6 mg/dL with 20% reduction was adopted in the present study. However, since there were no differences in frequency of CKD and baseline kidney function between groups, baseline kidney function is probably not a potential risk factor of AKI. Fourth, the rigorous gentamicin level for prevention of AKI may be less than 1 µg/mL. However, in the meta-analysis to assess gentamicin associated nephrotoxicity, due to the scarcity of suitable clinical trial, target gentamicin trough level below 1 µg/mL was not available [5]. Furthermore, since gentamicin associated AKI was multifactorial, the toxicity was caused by the administered duration, severity of illness as well as the levels [4, 19]. In the present study, physiological no differences of conditions, including qSOFA score, ejection fraction and kidney function, were observed between the two groups at baseline. Therefore, we adopted the gentamicin level less than 2 µg/mL as the threshold to mitigate remarkably higher exposure of gentamicin in this study. Lastly, the present analysis had a total timeframe of 23 years. Standard management for aminoglycosides had changed during the study period. Previous guidelines recommended administering gentamicin in 2 or 3 equally divided doses for the treatment of prosthetic valve endocarditis caused by *Staphylococcus species* [20]. However, in the current WikiGuideline of infective endocarditis, once daily administration of gentamicin as an adjunctive treatment is recommended, rather than multiple divided doses per day [21]. In this regard, there was no significant difference for development of AKI between once daily and multiple times administration of gentamicin with adherence of trough concentration below 1 µg/mL, and furthermore, approximately 25% patients who did not develop AKI had gentamicin of trough concentration, ranging 1–2 µg/mL a recent report [22]. Regardless of mitigation of the influence of AKI caused by higher gentamicin exposure, it is unclear that our findings are generalized in patients treated with concomitant vancomycin and gentamicin trough value between 1 and 2 µg/mL. In addition, this study could not be conducted using rigorous criteria of gentamicin less than 1 µg/mL due to retrospective preliminary design and the timeframe. However, while during the period of the present study, the target plasma concentrations of vancomycin and gentamicin and vancomycin AUC were managed according to the guidelines or reviews [2, 3], the dose of vancomycin and gentamicin had wide ranges.

Tangvichitrerk et al., recently, suggested that threshold of vancomycin AUC to prevent AKI was lower in patients treated with concomitant nephrotoxic agents compared to patients with no concomitant use nephrotoxins [7]. Obviously, further investigation is required in those patients. Despite these limitations, our findings support the hypothesis that vancomycin exposure with AUC 530 − 600 mg·h/L is potentially associated with AKI development in patients treated with vancomycin plus gentamicin in the setting of TDM.

## Conclusion

In patients treated with concomitant vancomycin and gentamicin with trough level below 1–2 µg/mL, vancomycin AUC 530 − 600 mg·h/L is associated with AKI risk.

## Electronic supplementary material

Below is the link to the electronic supplementary material.

Supplementary Material 1

## Data Availability

No datasets were generated or analysed during the current study.

## Abbreviations

AKI: Acute kidney injury
AUC: Area under the curve
CKD: Chronic kidney disease
CLIA: Chemiluminescent immunoassay
FPIA: Fluorescence polarization immunoassay
KIMS: Kinetic interaction of microparticles in solution
qSOFA: Quick Sequential Organ Failure Assessment
ROC: Receiver operating characteristic
TDM: Therapeutic drug monitoring
