# Supplementary material for: A preliminary retrospective study of the safety of Vancomycin area under the curve in patients treated with concomitant use of Vancomycin and gentamicin
Source: J Pharm Health Care Sci. 2025 Apr 14;11:32. doi: 10.1186/s40780-025-00438-1 (PMC11998355; doi:10.1186/s40780-025-00438-1)
Supplement: Supplementary file 1 — Supplementary Material 1 [file 40780_2025_438_MOESM1_ESM.docx]

**Supplemental materials**

*Data collection*

The clinical data included demographics, body weight measured before commencement of vancomycin therapy and at TDM of vancomycin and gentamicin, primary diagnosis, comorbidities, ejection fraction by transthoracic echocardiography, quick Sequential Organ Failure Assessment (qSOFA) score [1], baseline complete blood counts and blood chemistry (including albumin, blood urea nitrogen, creatinine, creatinine clearance), add-on treatment for infectious diseases, clinical signs and/or symptoms of exacerbation infectious diseases after the commencement of concomitant use vancomycin and gentamicin, date of death, vancomycin and gentamicin dose, concomitant nephrotoxic drugs defined as non-steroidal anti-inflammatory drugs (NSAIDs), angiotensin converting enzyme inhibitors or angiotensin receptor blockers (ACEIs/ARBs), and diuretics [2], TDM of vancomycin and gentamicin , and occurrence of AKI. Creatinine clearance was estimated by Cockcroft–Gault equation [3]. In patients who had interruption and resumption or multiple courses of vancomycin plus gentamicin therapy, the longer course of treatment was analyzed.

*Assessment of qSOFA score*

The qSOFA was scored according to published criteria [1]. Briefly, the presence of respiratory rate 22/min or more, systolic blood pressure 100 mmHg or lower, and altered mentation was given a score of 1 each. Altered mentation was defined as Glasgow Coma scale score lower than 15.

*Vancomycin administration and pharmacokinetic analysis*

Vancomycin was infused intravenously over 1 to 1.5 hours, at doses ranging from 10.6 to 46.2 mg/kg/day with dosing intervals ranging from 8 to 24 hours. A loading dose of 15 to 30 mg/kg was administered according to the decision of the attending physician. Doses of vancomycin were individualized based on patients’ renal function, aiming at achieving trough concentrations of 15‒20 μg/mL before December 31, 2021, and AUC_24h_ of 400‒600 mg·h/L thereafter, according to clinical practice guidelines.

Plasma vancomycin concentrations were measured 2 hours after completion of infusion and/or before the subsequent dose as necessary. In pharmacokinetic analysis,

the AUC of vancomycin was calculated *post hoc* using a web application, Practical AUC-guided TDM for vancomycin version 3.0b, based on the Bayesian method provided by the Japanese Society of Chemotherapy [4, 5].

*Gentamicin administration*

Gentamicin was infused intravenously over approximately 1 hour, at doses ranging from 0.8 to 4.6 mg/kg/day with dosing intervals ranging from 8 to 24 hours. Doses of gentamicin were individualized at the decision of the attending physician, depending on patients’ renal function and aiming at achieving trough concentrations below 1−2 μg/mL

before July 31, 2016, and below 1 μg/mL thereafter. Trough plasma gentamicin concentrations were measured before the subsequent dose.

*Vancomycin assay*

Plasma vancomycin concentrations were measured using a fluorescence polarization immunoassay (FPIA) (TDx, Abbott, Tokyo, Japan) before 31 October 2011, a chemiluminescent immunoassay (CLIA) (ARCHITECT iVancomycin Immunoassay; Abbott, Tokyo, Japan) between 1 November 2011 and 20 September 2019, and kinetic interaction of microparticles in solution (KIMS) (COBAS, Vancomycin III, Roche Diagnostic, Tokyo, Japan) thereafter. These three methods have been shown to give comparable results [6]. Blood samples obtained from patients were centrifuged and the separated plasma was used for the assay. The calibration ranges (lower and upper limits of quantification) were from 2.0 to 100.0 μg/mL for FPIA, from 3.0 to 100.0 μg/mL for CLIA, and from 4.0 to 80.0 μg/mL for KIMS. The precision of FPIA, CLIA and KIMS as assessed by intra- and inter-assay coefficients of variation was < 6.0%, < 10% and < 12%, respectively.

*Gentamicin assay*

Plasma samples obtained from patients were used for the assay. Plasma gentamicin concentrations were measured using a fluorescence polarization immunoassay (FPIA) (TDx, Abbott, Tokyo, Japan) before 2 October 2011, and an enzyme-multiplied immunoassay technique (EMIT) (Siemens Healthcare Diagnostics Inc., Tokyo, Japan) thereafter. The calibration ranges (lower and upper limits of quantification) were from 0.27 to 10.0 μg/mL for FPIA and from 0.25 to 10.0 μg/mL for EMIT. The precision of FPIA and EMIT as assessed by intra- and inter-assay coefficients of variation was < 3.4% and < 15%, respectively. These methods have been shown to give comparable results.

**References**

1. Singer M, Deutschman CS, Seymour CW, Shankar-Hari M, Annane D, Bauer M, Bellomo R, et al. The Third International Consensus Definitions for Sepsis and Septic Shock (Sepsis-3). JAMA. 2016;315:801‒10. https://doi: 10.1001/jama.2016.0287.
2. Lapi F, Azoulay L, Yin H, Nessim SJ, Suissa S. Concurrent use of diuretics, angiotensin converting enzyme inhibitors, and angiotensin receptor blockers with non-steroidal anti-inflammatory drugs and risk of acute kidney injury: nested case-control study. BMJ. 2013;346:e8525. https://doi: 10.1136/bmj.e8525.
3. Cockcroft DW, Gault MH. Prediction of creatinine clearance from serum creatinine. Nephron. 1976;16:31‒41. https://doi: 10.1159/000180580.
4. Oda K, Hashiguchi Y, Kimura T, Tsuji Y, Shoji K, Takahashi Y, et al. Performance of Area under the Concentration-Time Curve Estimations of Vancomycin with Limited Sampling by a Newly Developed Web Application. Pharm Res. 2021;38:637‒46. https://doi: 10.1007/s11095-021-03030-y.
5. https://antimicrobials.mipdapps.net/shiny/rstudio/vancomycinPAT4_0c/. Accessed 20, November, 2024.
6. Chen CY, Li MY, Ma LY, Zhai XY, Luo DH, Zhou Y, et al. Precision and accuracy of commercial assays for vancomycin therapeutic drug monitoring: evaluation based on external quality assessment scheme. J Antimicrob Chemother. 2020 Aug 1;75(8):2110‒19. https://doi: 10.1093/jac/dkaa150.
